# Study of New Glass–Ceramic and Dense Ceramic Containing Biogenic Hydroxyapatite

**DOI:** 10.3390/ma18133059

**Published:** 2025-06-27

**Authors:** Tina Tasheva, Albena Yoleva, Janna Mateeva, Hristo Georgiev

**Affiliations:** 1 Department of Silicate Technology, University of Chemical Technology and Metallurgy, 8 Kliment Ohridski blvd, 1797 Sofia, Bulgaria; yoleva@uctm.edu (A.Y.);; 2 Department of Industrial Safety, University of Chemical Technology and Metallurgy, 8 Kliment Ohridski blvd, 1797 Sofia, Bulgaria

**Keywords:** hydroxyapatite, glass ceramic, dense ceramic, biomaterials

## Abstract

A novel bioactive glass–ceramic was developed using biogenic hydroxyapatite (BHA) synthesized from *Rapana venosa* (Black Sea) shells and monocalcium phosphate monohydrate [Ca(H_2_PO_4_)_2_·H_2_O] via solid-state synthesis. The prepared batches were obtained by combining BHA with SiO_2_, B_2_O_3_, and Na_2_O, melted at 1200 °C and melt-quenched in water to form glass–ceramic materials. Dense biogenic hydroxyapatite-based ceramics were successfully sintered at 1200 °C (2 h hold) using a 25 mass % sintering additive composed of 35 mass % B_2_O_3_, 45 mass % SiO_2_, 10 mass % Al_2_O_3_, and 10 mass % Na_2_O. Structural characterization was carried out using X-ray diffraction (XRD), Fourier-transform infrared spectroscopy (FTIR), and scanning electron microscopy (SEM). The resulting materials consisted of a well-defined crystalline hydroxyapatite phase [Ca_10_(PO_4_)_6_(OH)_2_] alongside an amorphous phase. In samples with increased SiO_2_ and reduced B_2_O_3_ content (composition 3), a finely dispersed Na_3_Ca_6_(PO_4_)_5_ crystalline phase appeared, with a reduced presence of hydroxyapatite. Bioactivity was assessed in simulated body fluid (SBF) after 10 and 20 days of immersion, confirming the material’s ability to support apatite layer formation. The main structural units SiO_4_, PO_4_, and BO_3_ are interconnected through Si–O–Si, B–O–B, P–O–P, and mixed Si–O–Al linkages, contributing to both structural stability and bioactivity.

## 1. Introduction

The development of advanced materials for biomedical applications is among the most important problems faced by modern material science. More and more scientific developments are emerging in the field of biomaterials based on glasses, glass ceramics, and ceramics. Hydroxyapatite (HA) is a calcium phosphate bioceramic and is the main material for applications in bone replacement and exhibits biocompatibility, osteoconductivity, and bioactive behavior, being able to bond to the bone directly. Hydroxyapatite has been widely used in biomedical applications due to its similar composition to that of bone and teeth, and its excellent biocompatibility [1,2,3,4]. Different types of composites on the base of biogenic (BHA) and synthetic hydroxyapatite (SHA) and glass phase were studied and cited in the scientific literature. BHA is obtained from various biological sources like animal bones (bovine, porcine), fish scales, eggshells, and seashells. BHA has a crystallite size that is similar to that found in natural bone. BHA is highly bioactive, meaning it can stimulate biological responses, making it suitable for bone regeneration and other applications. BHA is environmentally friendly and cost-effective [5,6,7]. HA has been reinforced with glasses, glass–ceramics, and ceramics to improve its mechanical properties. One way to improve the properties of the HA ceramics is the addition of glass as a second phase. Glasses used to obtain composites with hydroxyapatite are on the base of the systems Na_2_O–CaO–SiO_2_, B_2_O_3_–Na_2_O–CaO–SiO_2_, Na_2_O–CaO–P_2_O_5_–SiO_2_, and others. It is known from the scientific literature that glass-reinforced HA shows greater bioactivity compared to commercial HA [8,9,10,11,12]. This occurs because silicate-based glasses release critical concentrations of ions (such as Si, P or Ca) in vivo, which are able to stimulate bone formation and play an important role both in angiogenesis and in neo-vascularization. The disadvantage of HA is its poor mechanical strength. HA is difficult to sinter and thus is mechanically weak. HA is the most thermodynamically stable calcium phosphate ceramic compound at the pH, temperature, and composition of the physiological fluid. Due to the chemical similarity between HA and mineralized bone of human tissue, synthetic HA exhibits a strong affinity to host hard tissues. The formation of a chemical bond with the host tissue offers HA a greater advantage in clinical applications over most other bone substitutes, such as allografts or metallic implants. To improve the mechanical properties of HA, appropriate sintering additives are also applied for dense samples [13,14,15]. The mechanical strength and fracture toughness of hydroxyapatite ceramics can be improved by applying different sintering techniques that include the addition of a low melting secondary phase to achieve liquid phase sintering for better densification, and the incorporation of sintering additives to enhance densification through grain boundary strengthening [16].

This paper aims to obtain new glass–ceramic and ceramic materials containing biogenic hydroxyapatite for potential application in dental and regenerative medicine.

## 2. Materials and Methods

Biogenic hydroxyapatite powder used in this study was prepared from Black Sea *Rapana venosa* shells and monocalcium phosphate monohydrate Ca(H_2_PO_4_)_2_·H_2_O by solid-state synthesis at 1180 °C. The synthesis method and characteristics of hydroxyapatite powder are described in our previous publications [17,18]. Samples marked from 1 to 3 in Table 1 were prepared by homogenization and the melting of chemical reagents as powdered quartz, H_3_BO_3_, Na_2_CO_3_, and NaNO_3_, and 30 mass % of BHA powder in a corundum crucible at 1200 °C and melt-quenched in water. For the production of dense ceramic samples (compositions 4 and 5 in Table 1), BHA powder in quantities of 50 mass % and 75 mass % and 50 mass % and 25 mass % were pre-melted and milled with glass powder containing 35 mass % B_2_O_3_, 45 mass % SiO_2_, 10 mass % Al_2_O_3_, and 10 mass % Na_2_O. Samples with dimensions of 3 × 0.5 cm were pressed at a pressure of 50 MPa on a hydraulic press and fired at 1200 °C with 2 h hold. For the analysis of the structure of the obtained samples, X-ray diffraction (DRON 3M diffractometer, Cu Ka radiation, wavelength 1.5418 Å, 28 mA current and 40 kV voltage) was used. The FTIR spectra were recorded in the 4000–400 cm^−1^ range by using FTIR spectrometer Varian 600-IR (Melbourne, Australia). The samples for these measurements were prepared in the form of KBr–disks. The precision of the absorption maxima was ±3 cm^−1^. The scanning electron microscope Carl Zeiss GmbH (Jena and Oberkochen, Germany), model EVO 10, brand ZEISS was used for the SEM and EDS (Oxford, UK) analysis. The densities of the glasses were measured by applying the Archimedes principle, using an analytical scale Mettler Toledo New Classic ME 104 (Greifensee, Switzerland) equipped with a density determination kit for solids using distilled water as the immersion liquid. For each glass composition, the density of at least three different samples was measured at least ten times. The bioactivity behavior of the studied BHAp-containing compositions was traced in a simulated body fluid (SBF) medium of pH 7.4 in a water bath at 37 °C for 10 and 20 days. Preparation of SBF is performed by following the Kokubo protocol [19]. SBF solution has ion concentrations nearly equal to human blood plasma and is buffered at pH 7.40 with 50 mM trishydroxymethylaminomethane and 45 mM hydrochloric acid at 36.5 °C.

## 3. Results and Discussion

**Figure 1:**
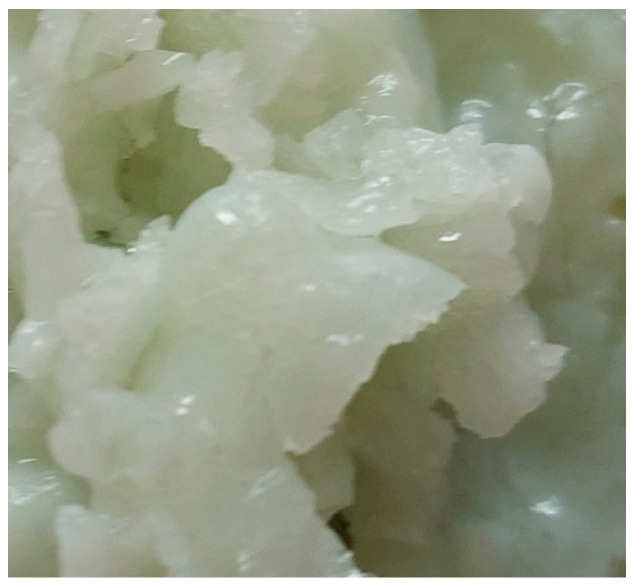
The sample with composition 3, melted at 1200 °C and melt-quenched in water.

**Figure 2:**
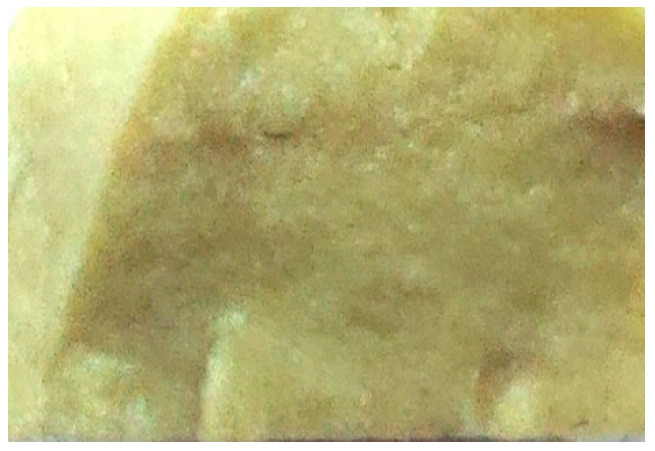
Sample with composition 5 pressed on a hydraulic press at 50 MPa and fired for 2 h at 1200 °C.

The results obtained show that samples with compositions from 1 to 4 are glass ceramics, and the sample with composition 5 is ceramic. In Figure 1 and Figure 2, the obtained samples with compositions 3 and 5 are shown. The results of the measured densities are presented in Table 1. The density of the glass melted samples increases with decreasing B_2_O_3_ content and increasing SiO_2_ content. The density of pressed ceramic samples increases with increasing hydroxyapatite content.

### 3.1. X-Ray Powder Diffraction

**Figure 3:**
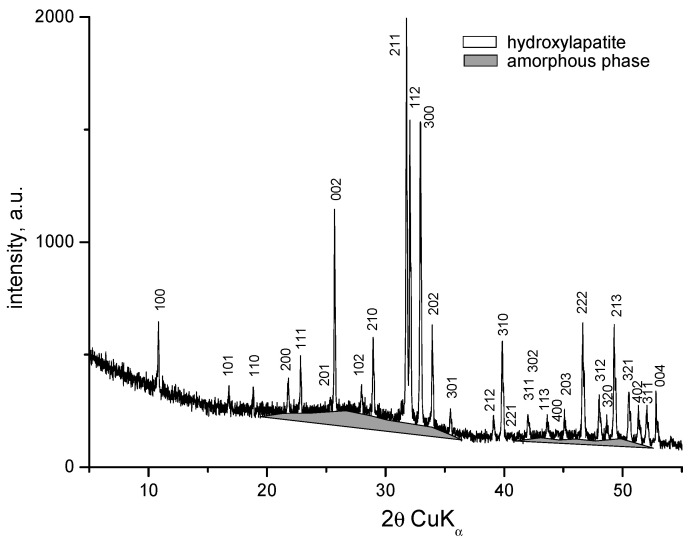
XRD patterns of the sample with composition 2 melted at 1200 °C and melt-quenched in water.

**Figure 4:**
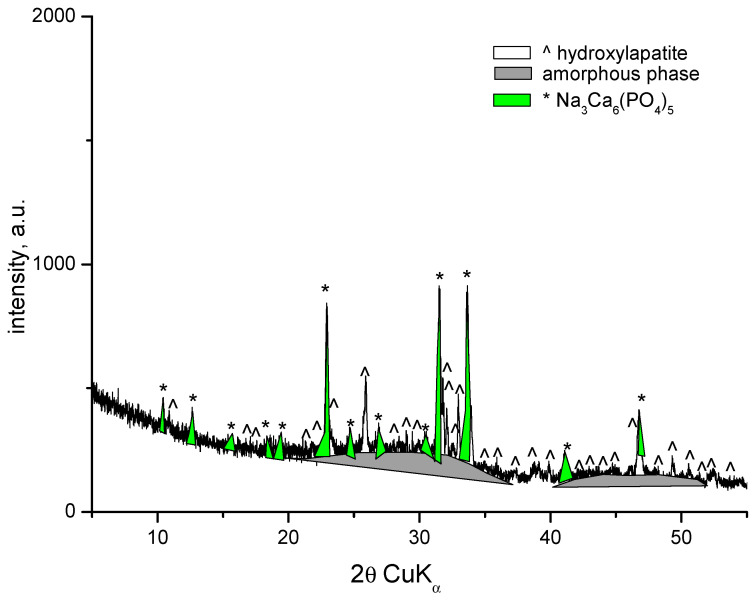
XRD of the sample with composition 3 melted at 1200 °C and melt-quenched in water.

XRD patterns of the samples with compositions 1 and 2 are identical and show amorphous halo and diffraction peaks. The XRD pattern of the sample with composition 2 is presented in Figure 3. Along with the amorphous phase, the main crystalline phase in both compositions is hydroxyapatite (PDF # 01-073-1731). The amorphous phase is more abundant in Sample 2. The X-ray diffractogram of the sample with composition 3 shows the presence of an amorphous phase, a small amount of hydroxyapatite (PDF # 01-073-1731), and the formation of a new fine crystalline phase of sodium calcium phosphate Na_3_Ca_6_(PO_4_)_5_ (PDF # 11-0236) (Figure 4). In this case, dissolution of hydroxyapatite in the amorphous phase and crystallization of sodium calcium phosphate probably occurs. H. Demirkiran et al. also identified the formation of the Na_3_Ca_6_(PO_4_)_5_ phase in hydroxyapatite–bioglass composites [20]. In compositions containing higher bioglass content (10 and 25 wt%), additional crystalline phases-namely calcium phosphate silicate (Ca_5_(PO_4_)_2_SiO_4_) and sodium calcium phosphate (Na_3_Ca_6_(PO_4_)_5_), were observed, embedded within the amorphous silicate matrices.

**Figure 5:**
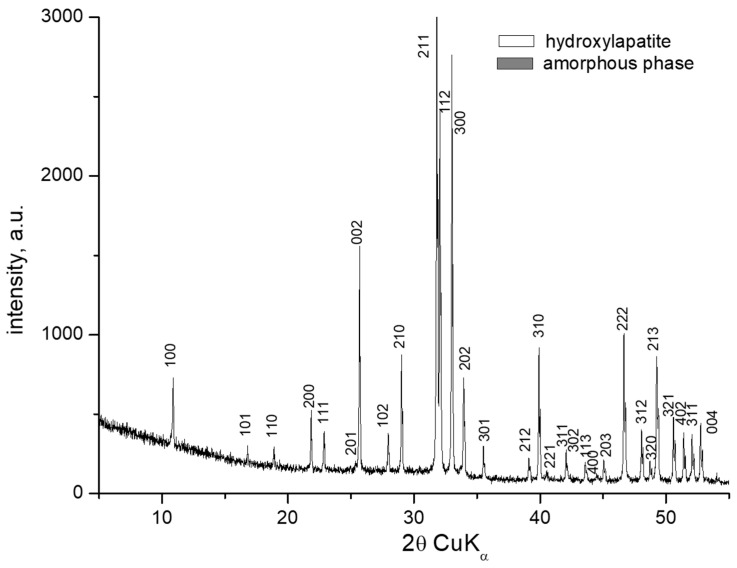
XRD of the sample with composition 5 pressed on a hydraulic press at 50 MPa and fired for 2 h at 1200 °C.

The diffractogram of the sample with composition 4 is similar to those of compositions 1 and 2, containing both hydroxyapatite and amorphous phases. In contrast, the diffractogram of the sample with composition 5 reveals only a hydroxyapatite phase (PDF # 01-073-1731) (Figure 5). Bioactive glasses have been successfully used as sintering additives to produce dense hydroxyapatite (HA) ceramics. These glasses typically contain components such as SiO_2_, B_2_O_3_, Na_2_O, K_2_O, Al_2_O_3_, P_2_O_5_, CaO, CaF_2_, MgO, and MgF_2_, among others [21]. Our results are consistent with data reported in the literature; in our case, borosilicate glass effectively facilitated the densification of HA ceramics through viscous flow, promoting liquid-phase sintering. Additionally, it stabilized the hydroxyapatite phase against thermal decomposition by encapsulating it within the glassy matrix.

### 3.2. FTIR Spectroscopy

**Figure 6:**
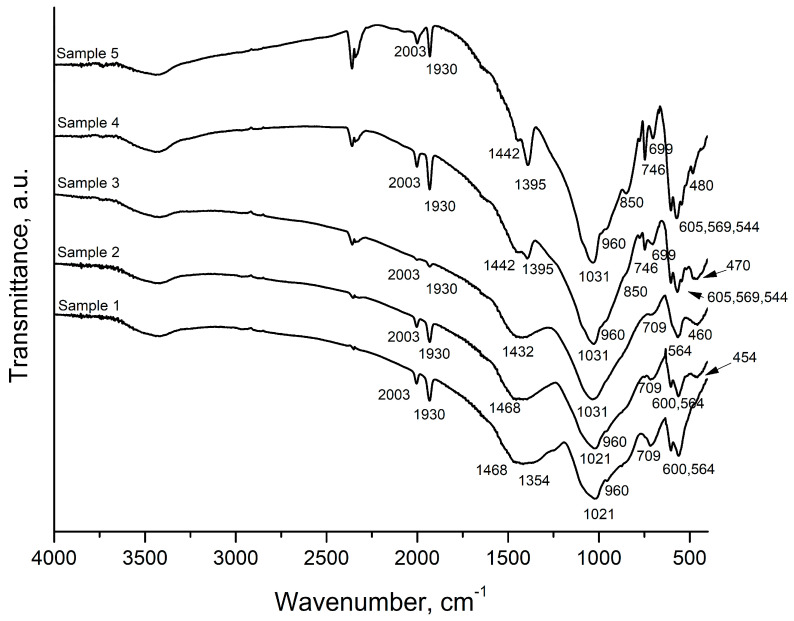
FTIR spectra of the samples.

The results of the FTIR spectra of the samples are presented in Figure 6. Several spectral ranges are observed high-frequency bands in the 1470–1354 cm^−1^ range, the 1021–1031 cm^−1^ range, the 700–750 cm^−1^ range, as well as bands at around 600 cm^−1^ and a band around 454–480 cm^−1^. The band at the high-frequency region becomes narrower and two well-defined bands appear at 1442 cm^−1^ and 1395 cm^−1^ in the spectra of Sample 4 and Sample 5. The band at 1021 cm^−1^ in the spectrum of Sample 1 and Sample 2 shifts to 1031 cm^−1^ in Samples 3, 4, and 5. A small shoulder at 960 cm^−1^ is observed in the spectra of all samples except Sample 3. The band at 709 cm^−1^ in the spectra of Samples 1 and 2 shifts to 699 cm^−1^ in the spectra of Sample 3, and a double degenerate vibration is observed in the spectra of Samples 4 and 5. The band at 564 cm^−1^ in the spectra of Sample 3 is double degenerated with bands at 600 cm^−1^ and 564 cm^−1^ in the spectra of Samples 1 and 2, and triple degenerated in the spectra of Samples 4 and 5 at 605 cm^−1^, 569 cm^−1^, and 544 cm^−1^. A band at 454 cm^−1^ with the addition of SiO_2_ in the spectra of Sample 2 appears; this band shifts up to 480 cm^−1^ in the spectra of Sample 5.

**Figure 7:**
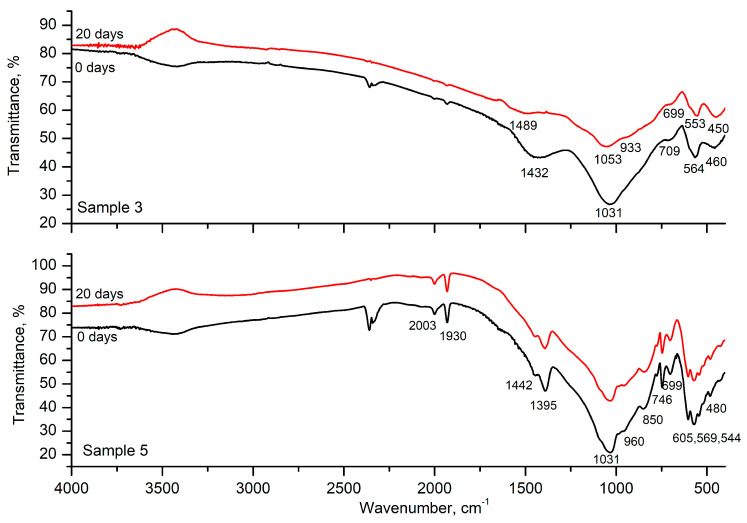
FTIR spectra of Sample 3: before and after 20 days in SBF (**top**) and Sample 5: before and after 20 days in SBF (**bottom**).

The results of the FTIR spectra of Samples 3 and 5 before and after 20 days in SBF are shown in Figure 7. The spectrum of Sample 3 after 20 days shows small changes. The bands at 1432 cm^−1^ and 1031 cm^−1^ are shifted to higher frequencies; a shoulder at 933 cm^−1^ appeared, and the bands at 709 cm^−1^, 564 cm^−1^, and 460 cm^−1^ are shifted to lower frequencies. No changes in the spectra are observed in the spectra of Sample 5 after 20 days in the SBF.

The structure of the glasses was studied by the FTIR spectroscopy (Figure 6). The vibrations of the isolated PO_4_ tetrahedra can be used as a starting point for the interpretation of phosphate spectra [16]. The isolated PO_4_ tetrahedra has four normal vibrations, namely a triple degenerative stretching antisymmetric vibration ν_d_^as^(F) at 1017 cm^−1^, a symmetric stretching vibration ν^s^(A_1_) at 938 cm^−1^, a triple degenerative bending δ_d_(F) at 567 cm^−1^, and a double degenerate deformation vibration δ_d_(E) at 420 cm^−1^. At the highest tetrahedral symmetry (Td), only the first and the third vibrations are active in the IR spectrum. As a result of a decrease in the symmetry of PO_4_ in the crystal structure, a degeneration of the degenerative vibrations and activation of the inactive vibrations occurs [22,23].

Koutsopoulos synthesized hydroxyapatite crystals from aqueous solutions using three approaches and summarized the analytical data for several calcium phosphates [23]. According to this study, the bands at 1087 cm^−1^, 1072–1032 cm^−1^, 962 cm^−1^, 601 cm^−1^, 571 cm^−1^, and 474 cm^−1^ are attributed to vibrations of the phosphate group, PO_4_. The peak at 1087 cm^−1^ in their spectra corresponds to the triple degenerate asymmetric stretching vibration, ν_3_. The other two components of this vibration appear at 1046 and 1032 cm^−1^. The peak at 962 cm^−1^ is assigned to the symmetric stretching mode, ν_1_, of the P–O bond in the PO_4_ group. The peaks at 601, 575, and 561 cm^−1^ are assigned to the O–P–O bond’s triple degenerated bending mode, ν_4_. The weak peaks at 472 cm^−1^ and the shoulder at 462 cm^−1^ are components of the double degenerated bending mode, δ_2_, of the phosphate group. The FTIR spectra of hydroxyapatite synthesized from Black Sea *Rapana venosa* shells by Yoleva et al. used in the present study also exhibit the previously mentioned bonds [17].

The addition of SiO_2_ causes a shift in the band at 1021 cm^−1^ to 1031 cm^−1^ and a new band at 454 cm^−1^ appears. Isolated SiO_4_ tetrahedra exhibit four normal vibrations [24]. However, only two of them, namely a triple degenerate asymmetric stretching vibration, ν_d_^as^(F), at 956 cm^−1^, and a triple degenerate bending vibration, δ(F), at 527 cm^−1^, remain active when the SiO_4_ tetrahedra have higher symmetry. It is found that while increasing the degree of linkage of tetrahedra in the order ortho-, pyro-, and meta- to disilicates, the position of the asymmetric stretching vibration ν_d_^as^(F) is shifted toward higher wavenumbers, and decreasing the symmetry of the tetrahedra induces an activation of the nonactive vibrations [24].

In the structure of borate materials, the boron atom can form coordination polyhedra as either BO_3_, BO_4_, or superstructural units built up of both BO_3_ and BO_4_ polyhedra [25,26]. The characteristic IR regions of vibrations of B–O bonds are related to the asymmetric B–O stretching vibration of borate tetrahedral (BØ_4_^−^) around ~800–1200 cm^−1^ range, the corresponding vibrations of boron oxygen triangular units (BØ_3_ and BØ_2_O^−^) at 1200–1650 cm^−1^, and the deformation vibrations of the network units give rise to weak absorption bands between 550 and 800 cm^−1^ [24].

Based on the data above, the following assignments of the bands that occurred in our spectra can be made (Table 2). The band at 1021 cm^−1^ can be assigned to the triple degenerate stretching antisymmetric vibration ν_d_^as^(F) of PO_4_, the band at 960 cm^−1^ to the symmetric stretching vibration ν_s_(A_1_) of PO_4_, and the doublet at 600 cm^−1^ and 564 cm^−1^ to the triple degenerate bending vibration δ_d_(F) of PO_4_ unit in the spectra of samples with composition 1 and 2. The degeneracy of the triple degenerative bending vibrations δ_d_(F) of the PO_4_ unit is observed in the spectra of the samples with compositions 4 and 5, where the triplet at 605 cm^−1^, 569 cm^−1^, and 544 cm^−1^ is observed. These spectral bands are characteristic of the hydroxyapatite phase. In the spectra of the sample with composition 3, the band related to the triple degenerate bending vibrations δ_d_(F) of the PO_4_ unit is represented by a singlet. This indicates the presence of a different symmetrical form of the PO_4_ unit. Such an observation follows the results obtained by XRD where a new crystalline phase appears (Na_3_Ca_6_(PO_4_)_5_).

The bands related to the triple degenerate stretching antisymmetric vibration ν_d_^as^(F) of SiO_4_ group overlap with those of PO_4_ in the high-frequency region. The bending vibrations of Si–O–Si of connected SiO_4_ units are found at 454 cm^−1^. However, a further increase in the SiO_2_ and hydroxyapatite content causes a shift in this band towards higher frequencies, where the double degenerated bending vibrations δ_d_(E) of PO_4_ and SiO_4_ tetrahedra are observed [22,23]. The peak at 2000 cm^−1^ is assigned to a 2.ν_3_ harmonic overtone or a combination mode, ν_1_ + ν_3_ [23]. The broad band at the 1468–1354 cm^−1^ region becomes narrower with decreasing B_2_O_3_ content and two well-defined peaks are formed in the spectra of Samples 4 and 5. These bands could be assigned to the overlapping vibrations of the stretching mode (ν_1_) of the CO_3_^2−^ group in A-type CAP or the bending mode (ν_4_ or ν_3_) of the CO_3_^2−^ group in A- and B-type CAP and the double degenerative asymmetric stretching vibration ν_d_^as^(E) of borate triangular units BO_3_ [24,26]. The band at 850 cm^−1^ could be assigned to the stretching vibrations of the CO_3_^2−^ group in CAP [23]. The degenerate bending vibration δ_d_(E) of BO_3_ units is at 560 cm^−1^. The band at 706–701 cm^−1^ could be assigned to the overlapping of bending vibrations of B–O–B in the [BO_3_] group and symmetric stretching vibrations of Si–O–Al [24,25].

### 3.3. SEM and EDS Analysis

**Figure 8:**
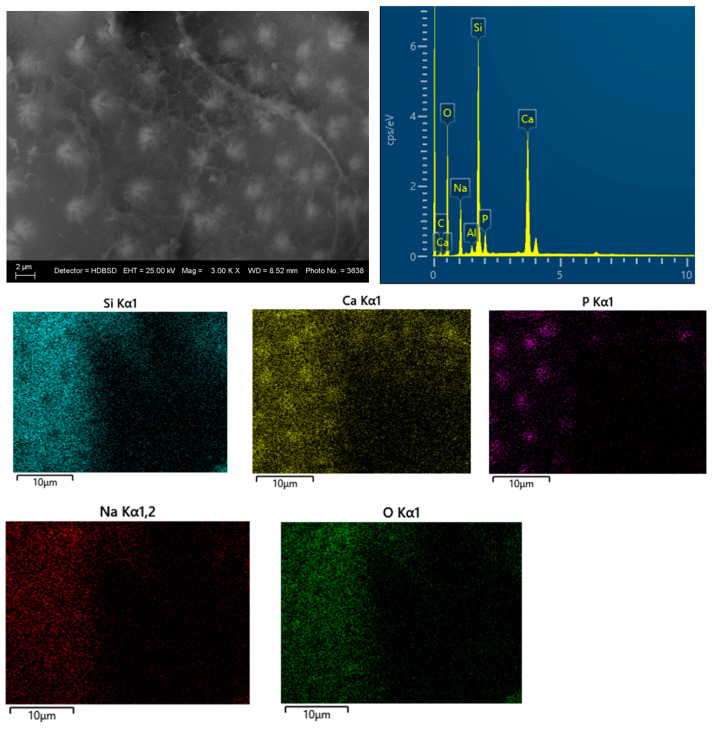
SEM and EDS of glass–ceramic sample with composition 3.

**Figure 9:**
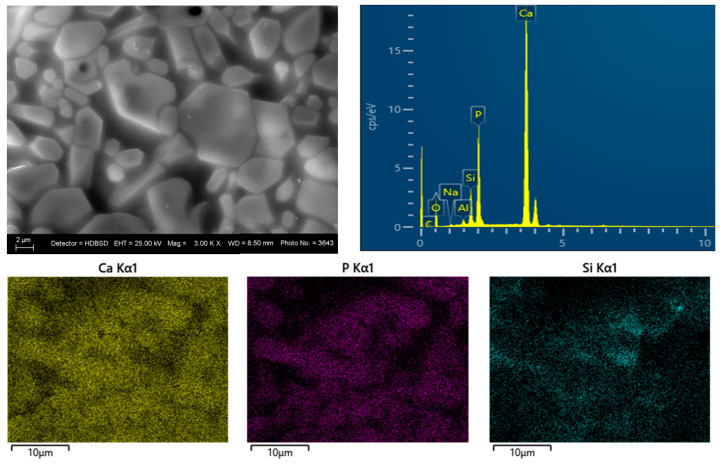

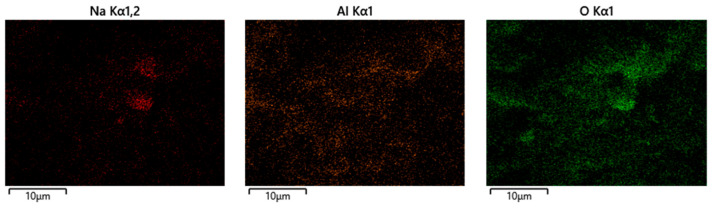
SEM and EDS of the ceramic sample with composition 5.

SEM and EDS analyses of the glass–ceramic materials with biogenic hydroxyapatite confirm the results of XRD and FTIR analyses. The results from SEM and EDS analyses of glass–ceramic samples with composition 3 are presented in Figure 8. Uniformly distributed hydroxyapatite and Na_3_Ca_6_(PO_4_)_5_ are observed in the amorphous matrix. SEM and EDS of ceramic samples with composition 5 are presented in Figure 9. The SEM image of the sample with composition 5 shows hexagonal crystals of hydroxyapatite with a size of 10 µm, sintered with the aid of the glass additive.

**Figure 10:**
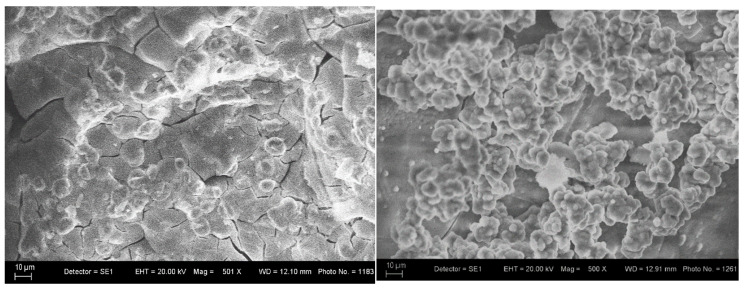
SEM glass–ceramic sample with composition 3 after immersion in SBF for 10 and 20 days.

**Figure 11:**
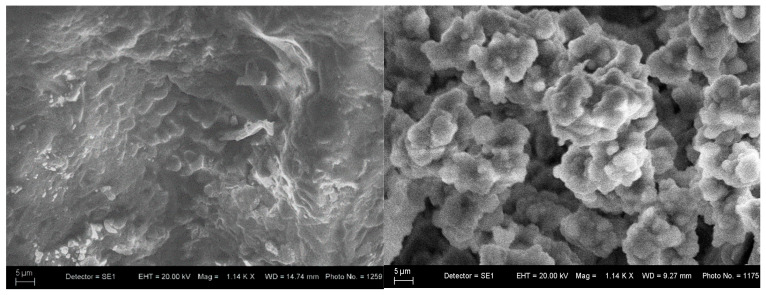
SEM of the ceramic sample with composition 5 after immersion in SBF for 10 and 20 days.

Figure 10 shows the SEM of the glass–ceramic sample with composition 3 after 10 and 20 days in the SBF solution. Figure 10 shows that the glass–ceramic with composition 3 has good bioactivity, since a new powdery layer of apatite is deposited on the glass–ceramic surface from the SBF solution after a stay of 20 days. The behavior of the hydroxyapatite ceramics (Composition 5, Figure 11) is similar. The SEM images of the glass–ceramic and hydroxyapatite ceramic (Figure 10 and Figure 11) illustrate the changes in surface morphology of the samples and hydroxyapatite crystals, which are covered by a layer from apatite after the soaking of the samples for 20 days in SBF solution.

The main glass-forming oxide in composition 1 is B_2_O_3_, and the main crystalline phase, which is observed, together with the amorphous one, is hydroxyapatite [Ca_10_(PO_4_)_6_(OH)_2_]. The addition of 20% by weight at the expense of B_2_O_3_ and Na_2_O does not result in a change in the phase (composition 2). In contrast, in composition 3 (45 wt% SiO_2_) the main phase is Na_3_Ca_6_(PO_4_)_5_. This shows that with the same amount of BHA, but with a change in the glass-forming oxide in glass–ceramics (compositions 1–3), the formation of two different crystalline phases is observed. Composition 3 has good bioactivity, since a new powdery layer of apatite is deposited on the glass–ceramic surface by the SBF solution after a stay of 20 days.

## 4. Conclusions

A new bioactive glass–ceramic was successfully developed by the melting at 1200 °C and the rapid quenching in water of batches combining 30 mass % biogenic hydroxyapatite (BHA), synthesized from *Rapana venosa* shells and monocalcium phosphate monohydrate [Ca(H_2_PO_4_)_2_·H_2_O] via solid-state synthesis at 1180 °C, and SiO_2_, B_2_O_3_, and Na_2_O. The resulting glass–ceramics contain both an amorphous phase and crystalline phases of hydroxyapatite [Ca_10_(PO_4_)_6_(OH)_2_] and a newly formed, finely dispersed Na_3_Ca_6_(PO_4_)_5_ phase. With an increase in the content of SiO_2_ at the expense of B_2_O_3_ (composition 3) the quantity of the finely dispersed Na_3_Ca_6_(PO_4_)_5_ crystalline phase increases and that of hydroxyapatite decreases as a result of the partial dissolution of hydroxyapatite into the glass matrix, followed by crystallization during cooling. After immersing for 20 days in simulated body fluid (SBF), SEM analysis confirmed morphological transformations and that an apatite layer forms on the surface of the glass–ceramic. The main structural units in the glass–ceramic—SiO_4_, PO_4_, and BO_3_—are interconnected via Si–O–Si, B–O–B, P–O–P, and mixed Si–O–Al bonds.

A dense hydroxyapatite ceramic was also produced using a glass-based sintering additive (35 mass % B_2_O_3_, 45 mass % SiO_2_, 10 mass % Al_2_O_3_, 10 mass % Na_2_O) to the biogenic hydroxyapatite. SEM analysis of composition 5 revealed well-formed hexagonal hydroxyapatite crystals (~10 µm). The borosilicate glass facilitated densification via viscous flow and stabilized the hydroxyapatite phase against thermal decomposition by encapsulating it in the glass matrix. After immersion in simulated body fluid (SBF) for 10 and 20 days, SEM analysis confirmed morphological transformations and the formation of an apatite layer on the ceramic surface, indicating bioactivity through ion exchange with the SBF.

Further structural, mechanical, and biological analyses will be performed. The resulting glass–ceramic containing BHA will be tested in future studies for potential application as a biomaterial in dental medicine. As for the sintered dense ceramic, our research will continue in the field of mechanical and biological studies of the resulting ceramic for its potential application in regenerative medicine.

## Figures and Tables

**Table 1:** Compositions of the studied biogenic hydroxyapatite samples.

Sample No.	Composition, Mass %				Density, g/cm^3^
	SiO_2_	Na_2_O	B_2_O_3_	BHA Powder	
1	-	20	50	30	2.48 ± 0.04
2	20	15	35	30	2.50 ± 0.04
3	35	10	25	30	2.54 ± 0.04
4	50 mass % powdered glass with the following composition in mass %: 35 B_2_O_3_, 45 SiO_2_, 10 Al_2_O_3_, 10 Na_2_O			50	2.60 ± 0.04
5	25 mass % powdered glass with the following composition in mass %: 35 B_2_O_3_, 45 SiO_2_,10 Al_2_O_3_, 10 Na_2_O			75	2.70 ± 0.04

**Table 2:** IR spectroscopy data for the samples, bands, and assignments.

Peak, cm^−1^	Assignment	References
2003, 1930	2.ν_3_ harmonic overtone or to a combination mode, ν_1_ + ν_3_	[23]
1468–1442	Stretching mode (ν_1_) of the CO_3_^2−^ group in A-type CAP or bending mode (ν_4_ or ν_3_) of the CO_3_^2−^ group in A and B-type CAP	[25]
1354–1395	Asymmetric B–O stretching vibration of borate triangular units BO_3_	[24]
1021–1031	Triple degenerate stretching antisymmetric vibration ν_d_^as^(F) of PO_4_	[17,22,23]
960	Symmetric stretching vibration ν^s^(A_1_) of PO_4_	[22,23]
850	Stretching mode of the CO_3_^2−^ group in CAP	[23]
709 699, 746	Overlapping of bending vibrations δ of B–O–B in [BO_3_] and symmetric stretching vibrations ν^s^ of Si–O–Al	[24,25,26]
564 600, 564 605, 569, 544	Triple degenerative bending δ_d_(F) of PO_4_	[17,22,23]
470–480	Overlapping of double degenerated bending vibrations δ_d_(E) of PO_4_ and SiO_4_ tetrahedra	[23,24]
454	Bending vibration δ of Si–O–Si of connected [SiO_4_] units	[24]

## Data Availability

The original contributions presented in this study are included in the article. Further inquiries can be directed to the corresponding author.
